# Overall survival following palliative immune checkpoint inhibitor treatment according to ECOG performance status: a large registry-based study

**DOI:** 10.2340/1651-226X.2026.45827

**Published:** 2026-07-03

**Authors:** Per Nodbrant, Eva Ulff, Magnus Lagerlund, Sander Ellegård, Olle Eriksson, Freddi Lewin, Kristina Engvall

**Affiliations:** a Department of Oncology, County Hospital Ryhov, Jönköping, Sweden; b Department of Oncology and Radiation Physics, Kalmar County Hospital, Kalmar, Sweden; c Department of Oncology, Linköping University Hospital, Linköping, Sweden; d Department of Biomedical and Clinical Sciences, Linköping University, Linköping, Sweden; e Futurum – the Academy for Health and Care, Jönköping, Sweden

**Keywords:** Immunotherapy, neoplasms, palliative treatment, ECOG performance status

## Abstract

**Background and purpose:**

Palliative immune checkpoint inhibitor (pICI) treatment has improved overall survival (OS) in many solid tumours during the last decades. However, the survival benefits are based on fit patients included in the clinical trials, all with Eastern Cooperative Oncology Group Performance status (ECOG PS) 0–1. Data on survival in PS 2–3 patients are limited.

This study aimed to compare OS according to ECOG PS at the initiation of pICI in real world data.

**Patient/material and methods:**

ICI has been recorded in the Swedish National Cancer Drug Registries since 2010. Palliative ICI treatment in the Southeastern region, Sweden, from 2010 until April 2025 were included. Survival probability and median overall survival (mOS) were calculated.

**Results:**

A total of 1956 patients had received at least one pICI treatment, for lung cancer (*n* = 854), melanoma (*n* = 443), renal carcinoma (*n* = 218) and urothelial carcinoma (*n* = 127). PS 2 or higher was reported in 28.9% and 80.6% received ICI only (no chemotherapy). mOS was in PS 0–1 patients 23.4 months, in PS 2 patients 9.6 months and in PS 3 patients 3.8 months. Death within 90 days occurred in 45.7% of PS 3 patients.

**Interpretation:**

Less fit patients are commonly treated with pICI, and PS seems an important predictive factor for palliative ICI regardless of cancer subtype. In our data, mOS is particularly short in PS 3 patients, suggesting to refrain from treatment. mOS is shorter in PS 2 patients than in PS 0–1, warranting individual considerations in treatment decisions. Better tools to guide treatment decisions for PS 2 patients are desirable.

## Introduction

The development of immune checkpoint inhibitor (ICI) treatment during the last decades has improved the prognosis of several solid tumours. However, the survival benefits observed in clinical trials are based almost exclusively on patients who are fit and fully active meeting the inclusion criteria of Eastern Cooperative Oncology Group performance status (ECOG PS) 0 or 1 [1]. In a meta-analysis of 60 phase II and III randomised controlled trials (RCT) of palliative immune checkpoint inhibitor (pICI) treatment only 51 out of 35,000 patients (0.001%) had a PS of 2 or higher. No difference in survival was found between PS 0 and PS 1, but due to the very small number of less fit patients included in the RCTs the effectiveness of pICI in patients with ≥ 2 could not be assessed [2]. Prospective randomised trials for patients not eligible for the pivotal trials have been requested although few studies have been conducted.

The ECOG PS scale dates back to 1982 but is a tool still routinely used by oncologists today. It was developed from the Karnofsky and Zubrod scales to assess patients’ ability to tolerate chemotherapy [1]. The scale has been criticised for being overly simplistic, as it does not distinguish between impairments due to musculoskeletal or organ dysfunction, nor does it account for cognitive factors [3] and it is associated with a high interobserver variability [4]. Nonetheless, in the context of chemotherapy, the association between poor PS, increased toxicity and reduced overall survival (OS) is well established [5]. Now we have a wide array of therapeutic options including ICI with a toxicity profile fundamentally different from chemotherapy [6, 7]. Limited research has been conducted to validate the use of ECOG PS in patients treated with ICIs; nevertheless, the ECOG PS 0–1 criterion remains a cornerstone for inclusion in all ICI trials.

Few prospective trials have been conducted in patients with PS ≥ 2. Single atezolizumab have shown efficacy in frail non-small cell lung cancer (NSCLC) patients compared to single agent chemotherapy, although a modest gain in median overall survival (mOS) of 1.1 months [8]. Retrospective studies have reported shorter survival in patients with PS ≥ 2, for example, in lung cancer showing a mOS of 3.3 months versus 13.4 months for those with PS 0–1 [9], in a urothelial cancer mOS of 7.2 months compared to 15.2 months [10] and lastly in a study across advanced solid tumours, mOS for PS ≥ 2 patients of 3.1 months, compared to 12.1 months (*p* < 0.001). PS was strongly associated with survival after adjustment for treatment delay, age, smoking status, and sex [11]. However, the available studies are limited both in sample size and in duration of follow up. ICIs may cause serious side effects and are among the most expensive treatments available; therefore, evaluating factors predictive of efficacy is of considerable importance. The aim of this study is to compare OS among patients with various solid tumours according to ECOG PS at the initiation of pICI therapy.

## Patients/material and methods

The study is a registry-based real world follow up study of OS after pICI. The hypothesis is that worse PS at treatment initiation is associated with shorter OS in patients treated with palliative ICI.

In Sweden, the use of ICI therapy has been recorded since 2010, first in the national registry ‘New drugs in cancer care’ and since 2018 in the regional Cancer Drug Registries that are connected in a national network. The study included all patients that in the Southeast region between 2010 and 24th of April 2025 who received at least one pICI treatment at the doctors’ discretion and were recorded in the Cancer Drug Registry. Patients treated with other drugs or ICI with curative or adjuvant indication were not included.

The registries utilise Swedish personal identification numbers, which encode sex and age. Diagnosis, ECOG PS, prescribed drug and if combination treatment, treatment intention, treatment period and reason for end of treatment are variables collected from medical records to the registries. ECOG PS ranges from PS 0 (asymptomatic and fully active) to PS 5 (death). PS 2 is defined as a symptomatic patient, spending less than 50% of waking hours in bed, being ambulatory and capable of self-care, PS 3 a symptomatic patient spending more than 50% of waking hours in bed or chair, but not bedbound, and capable of limited self-care [1]. Mortality dates are recorded in the population registry, which is linked to the Cancer Drug Register via the personal identification numbers.

The current population of the Southeast region is in Östergötland approximately 470,000, Jönköping 370,000 and Kalmar 245,000 inhabitants. Jönköping County has recorded treatments since 2010, Kalmar County since 2013, and Östergötland County (including Linköping University Hospital) since 2016. Kalmar ceased recording of lung cancer patients to the registry from September 2020 due to time constraints. According to the yearly report from the cancer drug registries, Jönköping reported 100.5 treatments per 100,000 inhabitants, Östergötland 96.3 and Kalmar 65.3. The average reported treatments in the 21 registries/regions of Sweden were 72.4/100,000 inhabitants (range 0–107.7) [12], thus Jönköping and Östergötland showed a high rate of registration.

### Statistical analysis

Median follow up time was calculated from initiation of the first pICI to censor date (database lock) 24th of April 2025. Patients with missing data on PS were not included in the analysis. Individuals with protected identities are not included in the Cancer Drug Register and migration to other countries accounted for loss to follow-up. mOS and comparisons between PS groups were calculated by Kaplain–Meier methodology and log-rank test. Differences in mortality within 30 and 90 days, respectively, from start of treatment were calculated using Fisher’s exact test. Statistical calculations were performed in SPSS 29, R version 4.4.1 (R Core Team (2026). R: A Language and Environment for Statistical Computing. R Foundation for Statistical Computing, Vienna, Austria. https://www.R-project.org) and Survminer (Kassambara, A. Survminer: Drawing Survival Curves using ggplot2 R package version 0.5.2 2026).

## Results

The registry included 1956 patients who had received at least one pICI treatment. The most common diagnosis was lung cancer (43.7%), followed by melanoma (22.6%) and renal carcinoma (11.1%). The majority of patients were male (55.9%) and aged between 60 and 80 years (range 17–93 years). PS 2 was reported in 494 patients (25.3%) and PS 3 in 70 patients (3.6%) (see Table 1). In renal carcinoma PS was missing in 45 cases due to the use of Heng criteria instead of ECOG PS when recorded in the Cancer Drug Registry.

**Table 1:** Study population.

Characteristics		*N*	%
Total study population		1956	100
County	Östergötland	892	45.6
	Jönköping	710	36.3
	Kalmar	354	18.1
Sex	Men	1094	55.9
	Women	862	44.1
Age at treatment start	< 40	53	2.7
	41–50	102	5.2
	51–60	247	12.6
	61–70	600	30.7
	71–80	749	38.3
	> 80	205	10.5
Diagnosis	Lung cancer	854	43.7
	Melanoma	443	22.6
	Renal carcinoma	218	11.1
	Urothelial carcinoma	127	6.5
	Head and neck cancer	96	4.9
	Esophagus/gastric cancer	84	4.3
	Colorectal cancer	46	2.4
	Liver/gallbladder/ duct c.	46	2.4
	Breast cancer	41	2.1
	Pancreatic cancer	1	0.1
PS ECOG at treatment start	PS 0	520	26.6
	PS 1	824	42.1
	PS 2	494	25.3
	PS 3	70	3.6
	PS 4	1	< 0.01
	Missing^a^	47	2.4
Year treatment start	2010–2015	39	2.0
	2016–2020	765	39.1
	2021	222	11.3
	2022	303	15.5
	2023	263	13.4
	2024	306	15.6
	1st January–17th of April 2025	58	3.0
Immunotherapy^b^	Pembrolizumab	750	38.3
	Nivolumab	596	30.5
	Nivolumab + Ipililumab	256	13.1
	Atezolizumab	195	10.0
	Cemiplimab	79	4.0
	Ipililumab	34	1.7
	Avelumab	32	1.6
	Durvalumab	14	0.7
Treatment^b^	Single ICI	1345	68.8
	ICI + Chemotherapy/TKI	355	18.1
	Dual ICI	232	11.8
	Dual ICI + Chemotherapy	24	1.2

Palliative immunotherapy treatment registered in the Southeast health care region, Sweden from 1st of January 2010 to 24th of April 2025. PS: Performance Status; ICI: immune checkpoint inhibitor; ECOG: Eastern Cooperative Oncology Group; TKI: Tyrosine kinase inhibitor.

a Missing PS: 45 patients with renal carcinoma, 2 with breast cancer;

b First treatment with palliative immune checkpoint inhibitor.

**Figure 1:**
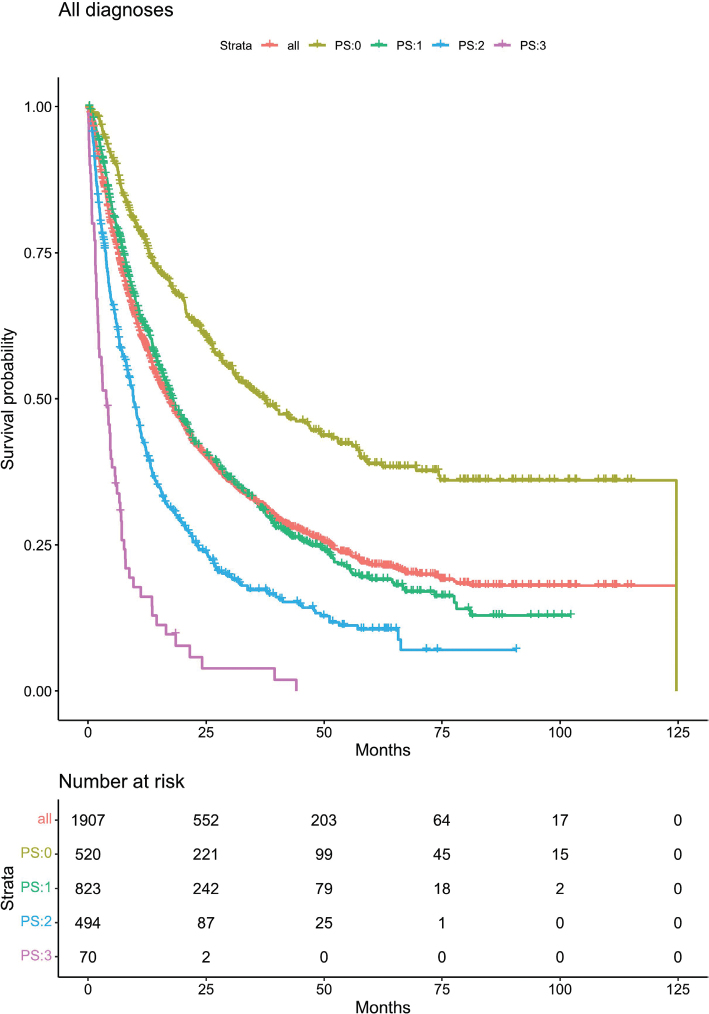
Overall survival probability by ECOG performance status (all respectively 0–3) at the first pICI treatment in the entire cohort (including all diagnoses, pICI single or combination) and treated 2010–24th of April 2025 in the Southeast region, Sweden and recorded in the cancer drug registry. pICI: palliative immune checkpoint inhibitor; ECOG: Eastern Cooperative Oncology Group; PS: performance status.

**Figure 2:**
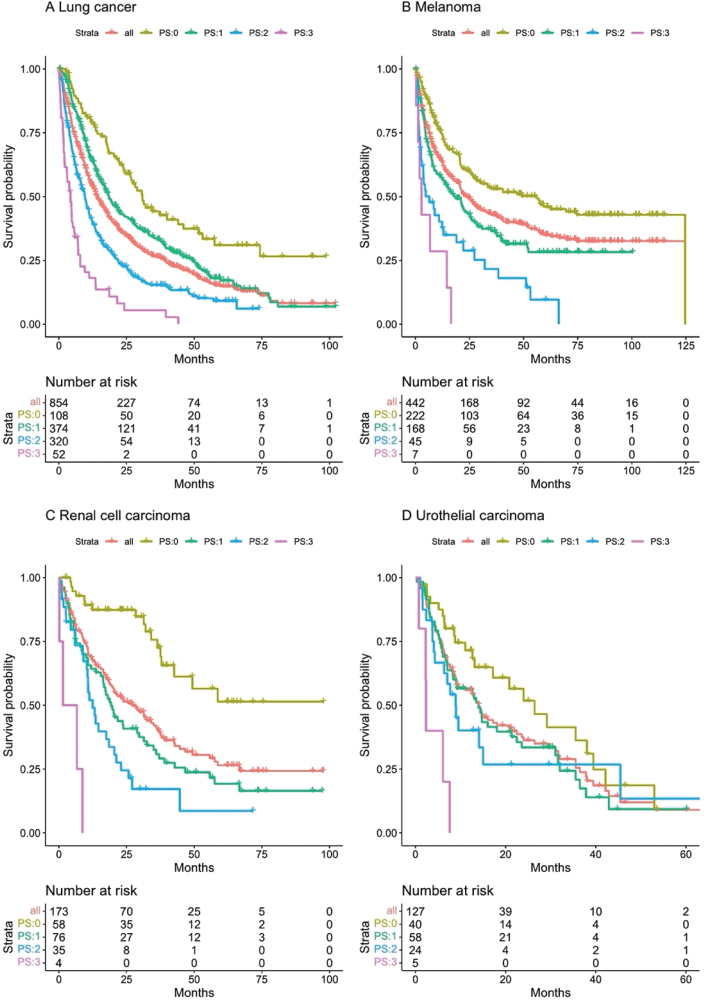
Overall survival probability by ECOG performance status at the first palliative immune checkpoint inhibitor treatment (single or combination), for (A) Lung cancer, (B) Melanoma, (C) Renal carcinoma and (D) Urothelial carcinoma treated 2010–24th of April 2025 in the Southeast region, Sweden and recorded in the cancer drug registry. ECOG: Eastern Cooperative Oncology Group; PS: performance status.

At the time of data extraction in April 2025, 35% of patients were alive. Median follow-up for all 1956 patients was 42.3 months ranging from 9.4 months for liver/cholangiocarcinoma to 57.2 months for melanoma. Poorer PS (defined as 2 or 3 compared to 0–1) was associated with shorter OS in the overall analysis (Figure 1), and also per diagnosis for lung cancer, melanoma, renal carcinoma, and urothelial carcinoma (Figure 2A–D) as well as for oesophageal-gastric carcinoma, although the latter included fewer than 100 cases. Colorectal and breast cancer had no registered cases of PS 3, but survival was shorter for PS 2 compared to 0–1. In urothelial carcinoma, head and neck cancer and liver- and cholangiocarcinoma no differences in OS were observed for PS 2 patients compared to 0–1, although numbers were low for these diagnoses (Table 2). Across diagnoses OS was better in men than in women and better in younger, aged 40–60 years compared to those over 60 years (data not shown).

**Table 2:** Median overall survival in months after start of the patients’ first palliative immune checkpoint inhibitor treatment based on diagnosis and ECOG Performance Status at treatment initiation.

Diagnosis	All PS mOS (95% CI)	PS 0–1 mOS (95% CI)	*N* (%)	PS 2 mOS (95% CI)	*N* (%)	P-value vs PS 0–1	PS 3 mOS (95% CI)	*N* (%)	*P*-value vs PS 0–1
Lung cancer	14.1 (12.4–15.8)	21.5 (17.5–25.4)	482 (56.4)	9.6 (8.1–11.2)	320 (37.5)	< 0.001	4.3 (2.5–6.1)	52 (6.1)	< 0.001
Melanoma	23.4 (18.3–28.4)	27.2 (18.0–36.4)	390 (88.2)	6.5 (1.1–11.8)	45 (10.2)	< 0.001	2.8 (0.6–5.1)	7 (1.6)	< 0.001
Renal carcinoma	27.1 (18.9–35.3)	36.0 (30.6–41.3)	134 (77.5)	12.5 (8.6–16.3)	35 (20.2)	< 0.001	1.5 (0.0–7.8)	4 (2.3)	< 0.001
Urothelial carcinoma	14.1 (10.3–17.9)	17.2 (9.9–24.5)	98 (77.2)	8.9 (6.2–11.6)	24 (18.9)	0.245	2.4 (2.2–2.5)	5 (3.9)	< 0.001
Head–neck cancer	15.8 (11.9–19.6)	16.8 (14.0–19.5)	61 (63.5)	11.6 (6.5–16.6)	35 (36.5)	0.706	N/A	0	N/A
Esophagus-gastric ca	10.8 (8.7–12.8)	13.3 (7.9–18.7)	63 (75.0)	8.4 (3.9–12.9)	19 (22.6)	0.005	0.1N/A	2 (2.4)	< 0.001
Liver, cholangio-carcinoma	18.2 (12.8–23.6)	18.2 (8.9–27.5)	43 (93.5)	24.4 N/A	3 (6.5)	0.778	N/A	0	N/A
Colorectal cancer	#	#	36 (78.3)	6.4 N/A	10 (21.7)	0.014	N/A	0	N/A
Breast cancer	16.6 (13.8–19.3)	16.6 (11.1–22.1)	36 (97.3)	3.3 (3.0–3.7)	3 (7.7)	0.046	N/A	0	N/A
All diagnosis	17.2 (15.6–18.8)	23.4 (20.8–26.0)	1343 (70.4)	9.6 (8.4–10.9)	494 (25.9)	< 0.001	3.8 (2.0–5.6)	70 (3.7)	< 0.001

PS: Performance status; ECOG: Eastern Cooperative Oncology Group; NA; Not Applicable.

# Median overall survival not reached. Pancreatic cancer *N* = 1 and PS 4 *N* = 1 (melanoma) were excluded due to low numbers. Median overall survival, standard error and confidence interval were calculated in SPSS 29. *P*-values for comparisons of survival curves among PS 0–1, PS 2 and PS3, respectively, were calculatd using the log-rank test.

Most patients (68.8%) received single-agent pICI, while 19.4% received single or dual pICI combined with chemotherapy or tyrosine kinase inhibitor (TKI) (see Table 1). Among those receiving combination therapy 38.8% were less fit; 133 with PS 2 and 14 with PS 3 (data not shown). The differences in OS according to ECOG PS were similar in the subgroup receiving palliative ICI as monotherapy without combination with chemotherapy; however, the mOS was generally longer in this subgroup than in the overall study population (Supplementary Figures 1 and 2A–D).

Early mortality occurring within 90 days of the first pICI dose was observed in a high proportion of PS 3 lung cancer, melanoma, and renal carcinoma patients (see Table 3). Among 52 lung cancer patients with PS 3, 21 (40.4%) died within 90 days; in melanoma and renal carcinoma 57% (4/7) and 50% (2/4) of patients, respectively, with PS 3 patients died within 90 days (see Table 3). Nevertheless, some PS 3 patients survived more than 1 year: seven (13%) with lung cancer and two (29%) with melanoma. Two lung cancer patients initiating treatment with PS 3 survived more than 3 years. Four of the 70 PS 3 patients were alive at the time of analysis (pICI started October 2023 or later; data not shown).

**Table 3:** Mortality within 30 and 90 days, respectively, after start of the patients’ first palliative immune checkpoint inhibitor treatment based ECOG performance status at treatment initiation.

Diagnosis	Total^b^ *N*	< 30 days *n* (%)	*P*-value^c^ vs PS 0–1	Total^b^ *N*	< 90 days *n* (%)	*P*-value^c^ vs PS 0–1
All diagnosis^a^						
PS 0–1	1339	19 (1.4)		1324	84 (6.3)	
PS 2	493	25 (5.1)	< 0.001	486	107 (21.6)	< 0.001
PS 3	70	14 (20.0)	< 0.001	70	32 (45.7)	< 0.001
Lung cancer						
PS 0–1	480	2 (0.4)		474	18 (3.8)	
PS 2	319	14 (4.4)	< 0.001	318	65 (20.4)	< 0.001
PS 3	52	10 (19.2)	< 0.001	52	21 (40.4)	< 0.001
Melanoma						
PS 0–1	387	12 (3.1)		385	37 (9.6)	
PS 2	45	3 (6.7)	0.198	44	17 (38.6)	< 0.001
PS 3	7	1 (14.3)	0.211	7	4 (57.1)	0.003
Renal carcinoma						
PS 0–1	134	2 (1.5)		132	7 (5.3)	
PS 2	35	2 (5.7)	0.190	33	6 (18.2)	0.025
PS 3	4	1 (25.0)	0.085	4	2 (50.0)	0.022

ECOG: Eastern Cooperative Oncology Group.

a Including the cancers below and breast cancer, pancreatic cancer, head and neck cancer, liver/cholangocarcinoma, esophagus-gastric carcinoma, colorectal cancer and urothelial cancer.

b Included cases with treatment start > 30 and > 90 days, respectively, before end of study date.

c Fisher’s exact test.

## Discussion and conclusion

In this study, registry-based cohort comprised of 1956 patients who initiated pICI treatment ECOG PS demonstrated a strong correlation with OS across various cancer subtypes. Individuals with PS 2 and especially those with PS 3 exhibited significantly reduced survival compared to patients with PS 0–1. Notably, mOS was substantially lower; from 23.4 months in patients with PS 0–1 to 3.8 months (*p* < 0.001) in those with PS 3.

For patients with PS 0–1, the mOS in our cohort was comparable to that reported in the registration trials that led to the approval of palliative ICI therapy. For example, mOS in our cohort of 18.5–30.8 months aligns with outcomes from RCTs in lung cancer, where mOS ranged from 11.8 to 22 months [13, 14]. These findings support the external validity of our cohort and suggest that outcomes among PS 0–1 patients that are treated in routine clinical practice approximate those seen in RCTs.

While pICI has transformed the prognosis for several cancers in patients with PS 0–1 [2], the benefit in PS 2–3 patients appears more limited. In our cohort mOS for patients with PS 3 was 2.8 months in melanoma and 1.5 months in renal carcinoma, compared with 27.2 and 36.0 months, respectively, in patients with PS 0–1. These findings mirror retrospective reports showing markedly poorer survival in patients with impaired PS [9–11]. For renal carcinoma, outcomes for patients with PS ≥ 2 in our material were consistent with those described by Khaki et al., who reported a mOS of 7.2 months [10]. A Danish registry study on melanoma patients who were categorised as ‘trial-excluded’, due to brain metastasis and/or PS ≥ 2, showed an increased survival in 2016 when ICI was available compared to 2012 without ICI treatment (mOS 6.9 vs. 4.2 months). In the trial-like population mOS in 2012 was 16.5 months and not reached 2016 [15]. Among patients with BRAF-mutated melanoma and a poor PS, treatment with BRAF/MEK inhibitors may be a more appropriate option [16].

The impact of PS on outcomes after pICI has been most extensively investigated in lung cancer. To our knowledge, only one phase III trial has included patients with PS ≥ 2: the Immunotherapy for Previously untreated, Suitability for Older/weaker and Single agent chemotherapy (IPSOS) study, which compared atezolizumab with vinorelbine or gemcitabine. As observed above, the trial demonstrated a modest improvement in survival with pICI in patients with PS 2–3 and/or older patients (> 70 years) with a mOS of 10.3 versus 9.2 months, although supported by favourable quality-of-life outcomes. However, no significant difference in mOS between pICI and chemotherapy was observed in the subgroup of 34 patients with PS 3 (*N* = 453) [8]. The differential impact of PS 2 versus PS 3 is rarely discussed in the literature, likely due to limited available data. In our study, we elected to separate these groups as PS ≥ 2 represents a heterogeneous population. For lung cancer, we observed a mOS of 9.6 months in patients with PS 2 – like results from the IPSOS trial – while patients with PS 3 had a mOS of 4.3 months. Both values were markedly lower than the mOS of 21.5 months observed in patients with PS 0–1.

A meta-analysis including 17,600 patients with lung cancer across interventional and observational studies, supplemented with previously unpublished extended data, has compared outcomes between PS 0–1 and PS ≥ 2. This analysis demonstrated a hazard ratio for OS of 2.76 (95% CI 2.43–3.14) for patients with PS ≥ 2 [17]. Similarly, a previous Swedish study of first line ICI for NSCLC stage IIIB–IV found for PS 2 mOS of 5.3 months in combination with chemotherapy and 5.0 months in monotherapy compared to 20.6 months and 19.8 months in PS 0–1 patients. However, mOS reached 27.3 months in PS 2 patients with non-squamous histology, programmed death-ligand 1 (PDL1) ≥ 50% and combination therapy, highlighting a wide range in mOS in PS 2 patients [18]. The single-arm PePS2 study, which evaluated pembrolizumab in patients with PS 2, reported survival outcomes similar to those observed in PS 0–1 patients in clinical trials, perhaps reflecting differences in patient selection relative to observational cohorts [19]. The management of patients with PS 2 has also been the subject of an expert consensus panel on the use of pICI in lung cancer, which concluded that PS 2 represents a negative prognostic factor and highlighted the need for greater inclusion of these patients in prospective clinical trials [20].

The registry data in our study showed that a limited number of patients with PS 3 were treated with pICI. Treatment decisions in these situations may be complex and in some patients PS 3 could be related to the tumour burden and pICI could be regarded as salvage treatment. However, for palliative patients with poor PS ≥ 2, key aspects are quality of life and treatment-related time toxicity. Bloom et al. reported that 50% of patients with PS ≥ 2 died within 30 days of initiating pICI, and that this group had substantially higher use of emergency department services, hospital admissions within the first months of therapy, and a greater likelihood of dying in hospital [21]. A landmark analysis in lung cancer similarly demonstrated that 86.4% of patients with PS 0–1 were alive at 3 months compared with 52.8% of those with PS ≥ 2, and at 12 months 41.0% compared with 13.4% [9]. In our cohort, we lack data on healthcare utilisation, but the finding that 46% of PS 3 patients died within 90 days strongly suggests limited benefit, potentially due to toxicities and/or rapid disease progression. As very few patients with PS ≥ 2 were included in the pivotal randomised trials, the true benefit of pICI in these individuals remains largely unknown for most cancer types. The IPSOS trial demonstrated only a modest difference in mOS, but its 2-year landmark analysis still showed a survival rate difference of 24% versus 12% favouring atezolizumab [8]. In this study, 13% of lung cancer patients with PS 3 were alive at 1 year, but we lacked a comparator arm. Despite this, the consistently short mOS observed in PS 3 patients raises the hypothesis that meaningful benefit from pICI is unlikely for most, which in turn has implications for health-economic evaluations within publicly funded healthcare systems. Overtreatment at the end of life poses ethical challenges, both in terms of patient burden and allocation of healthcare resources. Yet, the clinical reality is more complex: decisions must be individualised, balancing hope for response against the risk of harm. More robust predictive tools – beyond ECOG PS – are needed to support these conversations and guide decision-making.

Only 19.4% of patients in our cohort received combination therapy, mainly with chemotherapy and a few with TKIs. Analysis of OS in single agent pICI showed similar differences according to PS, alike other studies on pICI monotherapy for lung cancer with associations between ECOG PS ≥ 2 and poorer outcomes (14.3 vs. 6.8 months mOS) [22]. Elderly patients are also under-represented in trials, but similarly better PS (0–1) predicted longer OS from monotherapy pICI in older patients ≥ 70 years but was not predictive in younger patients [23].

Why patients with higher PS do not respond to immunotherapy to the same extent as fitter individuals remains largely unclear. A large tumour burden has been correlated to poor response to ICI and could correlate with higher PS status [24]. Although a correlation between immune-related adverse events and therapeutic efficacy is well established for checkpoint inhibitors, a lower frequency of such toxicities has been reported in less fit patients. It is, however, difficult to determine whether this reflects genuinely reduced immunological activation or simply shorter observation time due to early deterioration or death [22]. While the mechanisms underlying toxicity differ substantially between chemotherapy and pICI, it has been hypothesised that impaired immune competence may con tribute to the poorer outcomes observed in patients with reduced functional status [25]. Low physical activity has also been proposed to negatively influence immune function [26]. In lung cancer patients, additional hypotheses have focused on the use of corticosteroids that less fit patients may be more likely to require for symptom control. Similarly, antibiotic treatment – again possibly more frequent in patients with poorer PS – may disrupt the gut microbiome, which has been associated with impaired response to immunotherapy [22]. These factors could possibly contribute as discussed by Passaro et al, although definitive mechanistic explanations remain to be established [27].

Strengths of this study include the large population-based cohort encompassing multiple cancer diagnoses and institutions, which may reduce interobserver variability in PS assessment. The long median follow-up time together with complete mortality data obtained through linkage with national registries, enhances the robustness of the survival analyses. The use of real-world data further allows for the evaluation of outcomes in patient groups that are under-represented in clinical trials, providing insight into the effectiveness of pICI in routine clinical practice. Healthcare and access to pharmaceuticals are provided to all residents through a tax-funded welfare system, which minimises selection bias.

Nevertheless, several limitations should be considered. The retrospective design restricts the ability to infer causality, and the absence of a comparator arm prevents any definitive conclusions regarding treatment efficacy. We have no information on patients not selected for pICI, which could lead to an overestimation of mOS in PS ≥ 2 patients. In addition, data on prior systemic treatments, comorbidities, immune-related adverse events, healthcare utilisation, and health-related quality of life were not available in the registry. These missing variables may have influenced both treatment decisions and outcomes. Future research should aim to include patients with PS ≥ 2 in prospective trials and develop more refined tools for functional assessment – such as frailty indices – to better identify those PS 2 patients who may still derive meaningful benefit from pICI while avoiding overtreatment in those with very limited life expectancy.

PS appears to be a key predictive factor for patients receiving pICI therapy, irrespective of cancer diagnosis. Almost a third of all treated patients in the registry were PS ≥ 2, which emphasises clinical relevance of further prospective clinical trials for this large group of patients. In our data, mOS is particularly short in patients with a PS 3, suggesting refraining from treatment for these patients. mOS is substantially shorter in PS 2 patients than in patients with PS 0–1, warranting individual considerations in treatment decisions. More research is needed to further develop tools to guide treatment decisions for the PS 2 patients.

## Supplementary Material



## Data Availability

The data that support the findings of this study are derived from Swedish regional and national healthcare registries and are not publicly available due to legal and ethical restrictions, including data protection regulations. Aggregated data may be available from the corresponding author upon reasonable request, subject to approval by the relevant authorities.
