# Supplementary material for: Overall survival following palliative immune checkpoint inhibitor treatment according to ECOG performance status: a large registry-based study
Source: Acta Oncol. 2026 Jul 3;65:45827. doi: 10.2340/1651-226X.2026.45827 (PMC13343466; doi:10.2340/1651-226X.2026.45827)
Supplement: Supplementary file 1 [file AO-65-45827-s1.pdf]

Supplementary material has been published as submitted. It has not been copyedited, or typeset by Acta Oncologica

Supplementary Figure 1

Overall survival probability by ECOG Performance status (all respectively 0-3) at the first palliative immune checkpoint inhibitor treatment single agent without parallel chemotherapy in the entire cohort (including all diagnoses) and treated 2010-24th of April 2025 in the Southeast region, Sweden and recorded in the cancer drug registry.

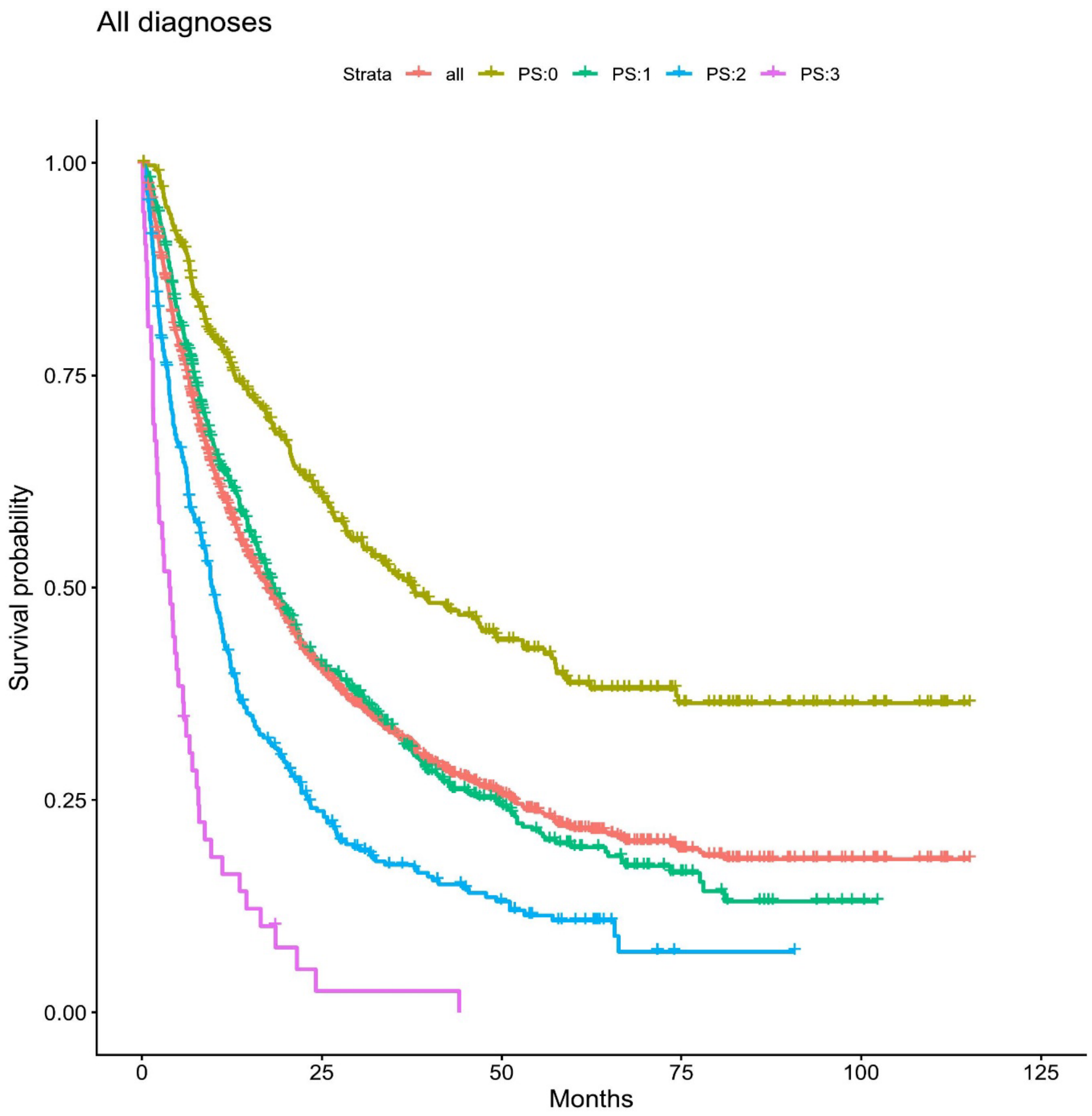

Number at risk

Strata	0	25	50	75	100	125
all	1351	426	177	60	16	0
PS:0	369	170	84	41	14	0
PS:1	578	187	68	18	2	0
PS:2	352	68	25	1	0	0
PS:3	52	1	0	0	0	0

Months

Supplementary Figure 2

Overall survival probability by ECOG Performance Status (all respectively 0-3) at the first palliative immune checkpoint inhibitor treatment single agent without parallel chemotherapy, for A) Lung cancer, B) Melanoma, C) Renal carcinoma and D) Urothelial carcinoma treated 2010-24th of April 2025 in the Southeast region, Sweden and recorded in the cancer drug registry.

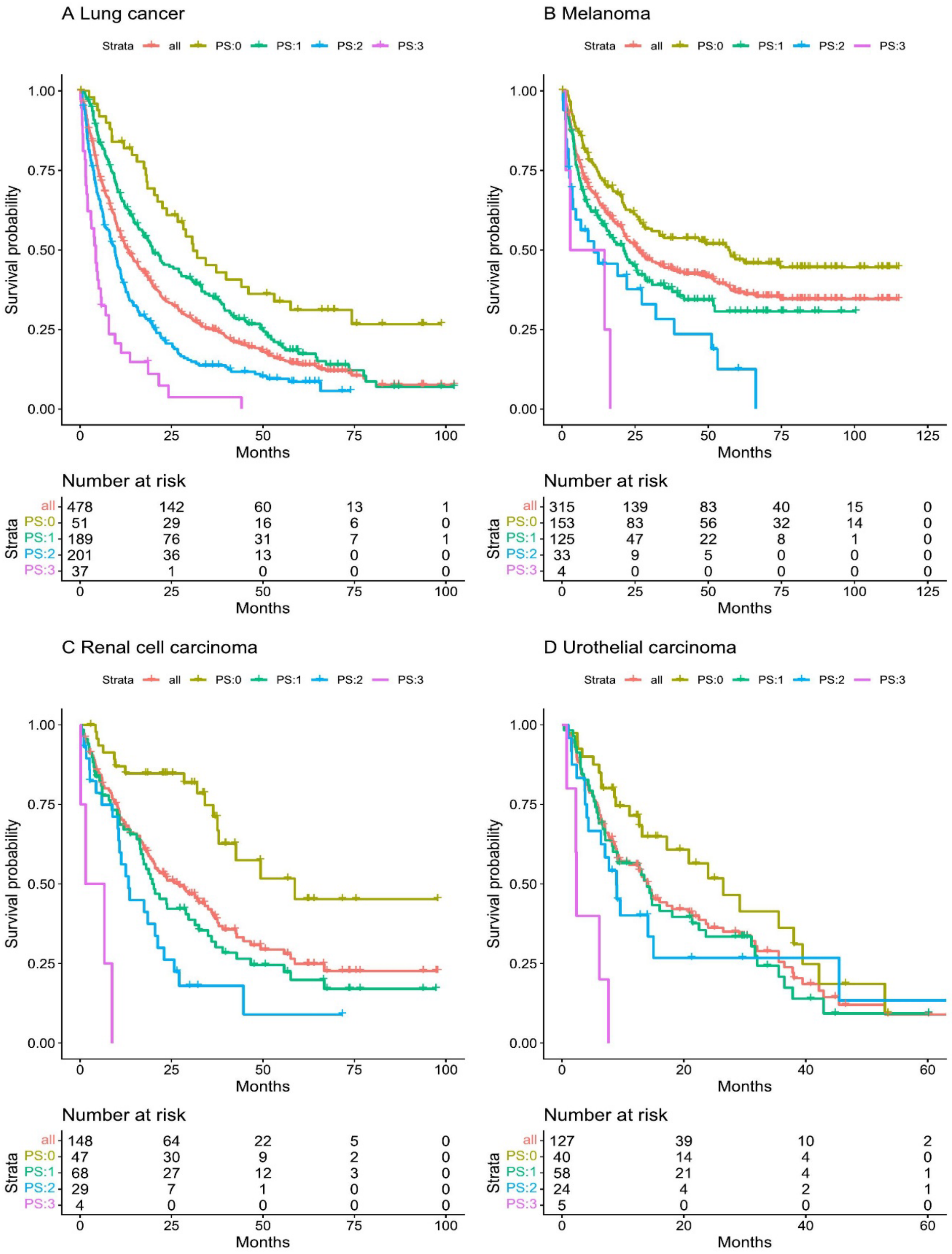
A Lung cancer
Strata all PS:0 PS:1 PS:2 PS:3
Survival probability
1.00
0.75
0.50
0.25
0.00
0 25 50 75 100
Months
Number at risk
all 478 142 60 13 1
PS:0 51 29 16 6 0
PS:1 189 76 31 7 1
PS:2 201 36 13 0 0
PS:3 37 1 0 0 0
Strata
0 25 50 75 100
Months
B Melanoma
Strata all PS:0 PS:1 PS:2 PS:3
Survival probability
1.00
0.75
0.50
0.25
0.00
0 25 50 75 100 125
Months
Number at risk
all 315 139 83 40 15 0
PS:0 153 83 56 32 14 0
PS:1 125 47 22 8 1 0
PS:2 33 9 5 0 0 0
PS:3 4 0 0 0 0 0
Strata
0 25 50 75 100 125
Months
C Renal cell carcinoma
Strata all PS:0 PS:1 PS:2 PS:3
Survival probability
1.00
0.75
0.50
0.25
0.00
0 25 50 75 100
Months
Number at risk
all 148 64 22 5 0
PS:0 47 30 9 2 0
PS:1 68 27 12 3 0
PS:2 29 7 1 0 0
PS:3 4 0 0 0 0
Strata
0 25 50 75 100
Months
D Urothelial carcinoma
Strata all PS:0 PS:1 PS:2 PS:3
Survival probability
1.00
0.75
0.50
0.25
0.00
0 20 40 60
Months
Number at risk
all 127 39 10 2
PS:0 40 14 4 0
PS:1 58 21 4 1
PS:2 24 4 2 1
PS:3 5 0 0 0
Strata
0 20 40 60
Months
